# Cuproptosis and cuproptosis–related genes in rheumatoid arthritis: Implication, prospects, and perspectives

**DOI:** 10.3389/fimmu.2022.930278

**Published:** 2022-08-04

**Authors:** Jianan Zhao, Shicheng Guo, Steven J. Schrodi, Dongyi He

**Affiliations:** ^1^ Department of Rheumatology, Shanghai Guanghua Hospital, Shanghai University of Traditional Chinese Medicine, Shanghai, China; ^2^ Guanghua Clinical Medical College, Shanghai University of Traditional Chinese Medicine, Shanghai, China; ^3^ Institute of Arthritis Research in Integrative Medicine, Shanghai Academy of Traditional Chinese Medicine, Shanghai, China; ^4^ Computation and Informatics in Biology and Medicine, University of Wisconsin-Madison, Madison, WI, United States; ^5^ Department of Medical Genetics, School of Medicine and Public Health, University of Wisconsin-Madison, Madison, WI, United States; ^6^ Arthritis Institute of Integrated Traditional and Western Medicine, Shanghai Chinese Medicine Research Institute, Shanghai, China

**Keywords:** rheumatoid arthritis, autoimmune disease, inflammation, cuproptosis, cuproptosis-related genes

## Abstract

Rheumatoid arthritis (RA) is an autoimmune disease that severely affects patients’ physical and mental health, leading to chronic synovitis and destruction of bone joints. Although various available clinical treatment options exist, patients respond with varying efficacies due to multiple factors, and there is an urgent need to discover new treatment options to improve clinical outcomes. Cuproptosis is a newly characterized form of cell death. Copper causes cuproptosis by binding to lipid-acylated components of the tricarboxylic acid cycle, leading to protein aggregation, loss of iron-sulfur cluster proteins, and eventually proteotoxic stress. Targeting copper cytotoxicity and cuproptosis are considered potential options for treating oncological diseases. The synovial hypoxic environment and the presence of excessive glycolysis in multiple cells appear to act as inhibitors of cuproptosis, which can lead to excessive survival and proliferation of multiple immune cells, such as fibroblast-like synoviocytes, effector T cells, and macrophages, further mediating inflammation and bone destruction in RA. Therefore, in this study, we attempted to elaborate and summarize the linkage of cuproptosis and key genes regulating cuproptosis to the pathological mechanisms of RA and their effects on a variety of immune cells. This study aimed to provide a theoretical basis and support for translating preclinical and experimental results of RA to clinical protocols.

## Introduction

Rheumatoid arthritis (RA) is an autoimmune disease characterized by chronic synovitis, presence of multiple autoantibodies, and bone and joint destruction (1). Genetic factors (common risk variants), environmental factors (smoking), genetic and environmental interactions (epigenetic mechanisms), and metabolic abnormalities are risk factors for RA (2). RA affects 1% of the global population and is more prevalent in women than in men (3). Current clinical treatment options for RA include disease-modifying anti-rheumatic drugs, non-steroidal anti-inflammatory drugs (NSAIDs), and biological and non-biological agents. Painkillers and NSAIDs reduce pain and stiffness, but NSAIDs have limited effectiveness and may cause stomach irritation, heart problems, and kidney damage (1). Disease-modifying antirheumatic drugs (DMARDs) are the primary treatment, and when used in combination, these drugs can slow the progression of RA and protect joints and other tissues from permanent damage. However, some DMARDs have multiple adverse effects, such as nausea, liver damage, bone marrow suppression, and development of lung infections (1). Biological agents, including anti-TNF-α antibodies, are also effective, but there are still adverse events, such as infection at the injection site and variation in the efficacy (1). In addition, proper lifestyle management, exercise, and food supplementation are prescribed as complementary therapies. However, due to multiple heterogeneous factors and a complex network of immune-inflammatory pathological mechanisms in RA, available therapies have shown limited clinical efficacy in some patients (2). Therefore, innovative discovery of new drug targets and elucidation of new mechanisms are of great importance for the clinical management of RA.

Cell death is closely associated with RA. Reduced apoptosis in fibroblast-like synoviocytes (FLS) leads to harmful and excessive proliferation, and other pro-inflammatory cell death mechanisms (e.g., pyroptosis and necroptosis) promote inflammation in RA (4). Tsvetkov et al. have characterized a novel form of cell death called “cuproptosis” (5). Cuproptosis in human cells occurs when mitochondrial respiration is disrupted, primarily by the direct binding of excess copper to the lipid-acylated components of the tricarboxylic acid (TCA) cycle. This leads to aggregation of lipid-acylated-related proteins, loss of iron-sulfur cluster proteins, and ultimately, cell death due to intracellular proteotoxic stress (5). In addition, Tsvetkov et al. identified 10 key genes for cuproptosis, including positive regulation factors (ferredoxin 1(*FDX1*), lipoic acid synthetase (*LIAS*), lipoyltransferase 1 (*LIPT1*), dihydrolipoamide dehydrogenase (*DLD*), drolipoamide S-acetyltransferase (*DLAT*), pyruvate dehydrogenase E1 subunit alpha 1(*PDHA1*), and pyruvate dehydrogenase E1 subunit beta (*PDHB*)) and negative regulatory factors (metal-regulatory transcription factor-1 (*MTF1*), glutaminase (*GLS*), and cyclin-dependent kinase inhibitor 2A (*CDKN2A*)) (5). A meta-analysis of 1444 patients with RA showed that their serum copper levels were significantly higher when compared to that of healthy controls (6). Similarly, Ma et al. found elevated serum copper and decreased zinc and selenium levels in RA patients by systematic evaluation and meta-analysis of common trace metals in them, with possible geographical differences in all three, and that serum selenium levels positively correlated with steroid treatment (7). In addition, serum copper levels were higher in patients with active RA, positively correlated with erythrocyte sedimentation rate (ESR) and morning stiffness, and negatively correlated with hemoglobin levels, which are auxiliary markers for disease assessment (8). Therefore, given the excess copper levels in RA, we sought to elucidate its potential association with RA by searching for cuproptosis and cuproptosis–related genes in *PubMed* to provide theoretical references and guidance for the discovery and innovative development of clinical treatment options for RA.

## Relationship between cuproptosis and RA

Tsvetkov et al. characterized an extensive and detailed characterization of cuproptosis (5). First, the factors necessary for cuproptosis include the presence of glutathione, and the mitochondrial metabolism of galactose and pyruvate (5). Second, cuproptosis appears to be more dependent on mitochondrial respiration, which is inhibited under various conditions such as hypoxia, and presence of mitochondrial antioxidants, inhibitors of mitochondrial function and fatty acids (5). Finally, the knockdown of seven genes that positively regulate cuproptosis may inhibit cuproptosis. For example, knockdown of *FDX1* results in the loss of protein-lipid acylation, decreased mitochondrial respiration, accumulation of pyruvate and α-glutarate, and loss of iron-sulfur cluster proteins (5). In addition, accumulation of regulatory gene oligomers is important for the occurrence of cuproptosis (5). Synovial tissue of patients with RA presents a hypoxic environment due to chronic inflammation, vascular proliferation, and excessive cell proliferation (9). Under hypoxic conditions, multiple mediators of bone destruction (matrix metallopeptidases (MMPs)), pro-inflammatory factors (interleukin 8 (*IL-8*) and *IL*-*6*), and chemokines (chemokine (C-C motif) ligand 20 (*CCL20*)) are involved in bone destruction and inflammatory processes in RA (10, 11). Multiple cells in RA are characterized by an imbalance between cell survival and cell death. The metabolic mechanisms associated with cuproptosis may be linked to these cells. For example, the overall glucose and glutamine levels were reduced in RA FLS, showing enhanced depletion, and indicating that glutamine plays an essential role in FLS proliferation. Glutamine is a critical factor in cuproptosis and its reduced levels, which lead to significant inhibition of cuproptosis, may contribute to the abnormal proliferation of FLS. RA FLS have multiple tumor-like features and survive and over proliferate in a tumor-like microenvironment. The aberrant proliferation of RA FLS is partially attributed to the inhibition of apoptosis (12, 13). The hypoxic environment may also inhibit cuproptosis and thus may contribute to abnormal cell survival and proliferation. The link between copper and hypoxia is complex. Hypoxic conditions promote copper cytotoxicity by inhibiting antioxidant defense mechanisms by increasing reactive oxygen species (ROS), copper transport, and mitotic phagocytosis, with specific molecular mechanisms possibly involving MTF1 and the forkhead box O-3 (FoxO3) signaling pathway (14). Additionally, similar to the inhibition of cuproptosis by the glycolytic effect of FLS, effector T cells exert their effect through the mTOR-dependent pathway, using glycolysis to take in large amounts of glutamine and glucose to provide energy, which may also inhibit cuproptosis, thereby exerting a pro-inflammatory effect. Overactivation of the glycolytic pathway may also inhibit Treg cell function (15). Activated M1 pro-inflammatory macrophages are glycolytic and release pro-inflammatory mediators through multiple mechanisms to destroy tissues (15). These factors may promote inflammatory effects by inhibiting the cuproptosis process in pro-inflammatory cell populations (**Table 1**). Next, we describe the potential association between critical genes associated with cuproptosis and RA development.

**Table 1:** The potential function of cuproptosis-related genes in RA.

Gene	May affect cells in RA	Function
*PDHA1*	FLS, Macrophages	PDHA1 inhibition may contribute to the FLS hyperproliferative state. PDHA1 may synergize with STAT3 to regulate the macrophage inflammatory response.
*PDHB*	Treg cell, FLS,	PDHB may co-regulate Treg cells and maintain functional integrity with DJ-1. Downregulation of PDHB may contribute to the abnormal proliferative state of RA FLS
*GLS*	FLS, CD4+T cell (Th1, Th2, Th17), B cell	GLS1 may promote aberrant proliferation of RA FLS, and GLS1 inhibition has different effects on different CD4+ T cell subpopulations. GLS is involved in regulating B cell activation and antibody production.
*LIAS*	Treg cell	LIAS is mainly involved through the regulation of oxidative stress and inflammation and has potential links to RA.
*DLAT*	FLS	DLAT may influence the development of RA mainly by affecting pyruvate oxidation in the PDHC, TCA cycle, and mitochondrial function
*FDX1*	Dendritic cells, monocytes-macrophages, Treg cells	FDX1 mainly affects fatty acid oxidation and steroid regulation, affecting different cells.
*MTF1*	FLS, T cells	MFT1 stimulates FLS recruitment and inflammatory factor production, promotes angiogenesis, and facilitates pro-inflammatory T cell arrest in the joints.
*CDKN2A*	Macrophages, T cells, B cells, FLS	CDKN2A is a marker of cellular senescence and may be involved in the aberrant proliferation of FLS and regulation of inflammatory factor release, promoting pro-inflammatory responses in monocytes and macrophages, and may be involved in the functional regulation of abnormal T and B cells.
*LIPT1*	FLS	LIPT1 is mainly responsible for regulating glutamine metabolism aiming to support mitochondrial respiration, TGA cycling, and fatty acid production, which may promote the abnormal proliferative process of FLS.

### PDHA1

Lactate levels are significantly increased and glucose concentrations are significantly decreased in RA synovial membranes, suggesting excessive activation of glycolytic pathways (16). Glycolysis converts glucose to pyruvate, and the downstream pathways of glycolysis include lactate fermentation and oxidation of pyruvate (17). *PDHA* has been extensively studied in tumor cells. Tumor cells promote their growth primarily by enhancing the glycolytic pathway and attenuating oxidative phosphorylation, which appears to also like the excessive glycolysis in RA FLS. During oxidative phosphorylation, the pyruvate dehydrogenase complex (PDHC) converts pyruvate to acetyl coenzyme A. *PDHA1*, a subunit of PDHC, is a key component linking glycolysis and the TCA cycle (18). *PDHA1* inhibition affects PDHC activity, leading to tumor cell glycolysis, enhanced consumption of glucose and glutamine, and inhibition of oxidative phosphorylation (19). Gut microbial-derived butyrate inhibits sirtuin 3 and mitochondrial complex I in tumor cells to prevent the conversion of TCA cycle intermediates to adenosine triphosphate (ATP). Butyrate induces hyperacetylation of PDHA1 to relieve the inhibition of PDHA1 phosphorylation at serine 293 to promote tumor cell apoptosis (20). The transcription factor RUNX family transcription factor 2 (*RUNX2*) promotes the expression of several glycolytic proteins (phosphorylated protein kinase B (*PKB*), hexokinase 2 (*HK2*), and PDH kinase 1(*PDHK1*)), inhibits the expression of PDHA1 and sirtuin 6 (*SIRT6*), and suppresses the rate of mitochondrial oxygen consumption (a marker of mitochondrial oxidative phosphorylation), thereby promoting tumor cell proliferation (21). Therefore, it can be speculated that *PDHA1* may be involved in RA FLS by regulating the glycolytic process. PDHA1 in RA FLS may be in an inhibited state, thus contributing to the excessive glycolytic and hyperproliferative state of FLS.

In addition to what has been described above, *PDHA1* can also be potentially linked to RA through the regulation of inflammation. The release of the NLRP3 inflammasome and related pro-inflammatory mediators plays an important role in the inflammation in RA (4). Activation of the nucleotide-binding oligomerization domain (NOD)-like receptor pyrin domain containing 3 (*NLRP3*) inflammasome requires lactate fermentation and inhibition of PDHA1 leads to impaired pyruvate oxidation. NLRP3 inflammasome activation leads to release of *IL-1β* pro-inflammatory mediators (17). Macrophages are important effector cells that are involved in the inflammatory response to RA. Macrophage *SIRT-3* is deacetylated at lysine 83, which activates PDHA1, and inhibits NLRP3 inflammasome activation and IL-1β release (22). In addition, the LPS-induced *in vitro* cell model is an important model for RA inflammation (23). Melatonin receptor 1 (MT1) inhibits LPS-induced aerobic glycolysis and impairs oxidative phosphorylation by promoting PDHA1 expression to suppress inflammation (24).The role of MT1 has been extensively studied in RA. MT1 plays critical roles such as altering the *Th1*/*Th17* balance to suppress inflammation (25) and reducing inflammation and cartilage degradation through the phosphatidylinositol 3−kinase (*PI3K*)/protein kinase B (*AKT*), extracellular signal-regulated kinase (*ERK*), and nuclear factor-κB (*NF-κB*) signaling pathways, as well as tumor necrosis factor α (*TNFα*) and *IL-1β* (26). In summary, PDHA1 appears to be a potent regulator of excessive glycolysis and inflammation and is regulated by different transcriptional mechanisms. Further studies specific to RA are still needed.

### PDHB

*PDHB* is a subunit of pyruvate dehydrogenase, which is similar in function to *PDHA1* in that they both catalyze pyruvate to acetyl coenzyme A (27). *PDHB* has been identified as a susceptibility gene for RA and its expression is downregulated in various tissues and cells (28). Deglycase DJ-1 was found to bind PDHB in Tregs, inhibit PDHA phosphorylation, and promote PDH activity and oxidative phosphorylation to maintain Treg cell differentiation and the functional integrity of T cells (29). In addition, *PDHB* has also been studied in various tumor cells. As previously mentioned, it may be linked to abnormalities in RA FLS. Maternally expressed gene 3 (*MEG3*) inhibits miRNA (miR)-103a-3p, upregulates *PDHB*-induced endoplasmic reticulum stress proteins’ expressions (glucose-regulated protein 78 (GRP78), activating transcription factor 6 (ATF6), C/EBP homologous protein (CHOP), caspase-3, and caspase-9), inhibits cell viability, colony formation ability and invasion, blocks the cell cycle, and induces apoptosis in tumor cells (30). MiR-203, miR-146b-5p, and miR-363-3p promote pro-tumor cell growth, invasion, inhibition of apoptosis, and enhancement of glycolysis by targeting *PDHB* (31–33). *PDHB* also inhibits *RasV12*-driven *ERK* signaling and tumor cell proliferation (34). The interaction between *PDHB* and NIMA-related kinase 10 (*NEK10*) may be necessary for maintaining mitochondrial homeostasis, and *NEK10* knockdown leads to increased mitochondrial damage and dysfunction (35). Thus, *PDHB* appears to be regulated by multiple miRNAs, while abnormalities in multiple miRNAs contribute to the pathological progression of RA, and the interconnection between the two deserves further exploration (36). In conclusion, downregulation of PDHB may contribute to the abnormal proliferative state of FLS in RA and may lead to defective Treg function through reduced binding to DJ-1.

### GLS

*GLS* primarily includes two isoforms, *GLS1* and *GLS2*, which are the key enzymes for glutamine metabolism. *GLS1* exists in two splice variants: *KGA *and *GAC* (37). GLS1 may promote abnormal proliferative processes in RA FLS. In response to the inflammatory factor *IL-17*, the mRNA expression of *GLS1* was upregulated, whereas the expression of *GLS2* was extremely low, implying that *GLS1* is primarily responsible for glutamine metabolism. Furthermore, the inhibition of *GLS1* suppresses the proliferation of RA FLS and improves joint inflammation in arthritic mice (38).

*GLS1* inhibition has multiple effects on CD4+ T cells and their subpopulations. First, it leads to α-CD3/CD28-induced suppression of CD4+ T cell proliferation and decreased expression of T cell activation markers CD25 and CD226 (39). Second, it inhibits cytokine secretion from multiple CD4+ T cell-differentiated T cell subsets, e.g., *IL-2* and interferon gamma (*IFN-γ*) (Th1 cytokines), *TNF-α*, *IL-6*, *IL-4* (Th2 cytokines), and *IL-17a* (Th17 cytokines) (39). Finally, the percentage of CD4+ T cells expressing chemokine (C-C motif) receptor 6 (*CCR6*) and C-X-C chemokine receptor 3 (*CXCR3*) is reduced (39), both of which have essential roles in inflammatory chemotaxis in RA (40, 41). Th17 is a critical pro-inflammatory mediator in RA that releases IL-17 pro-inflammatory factors to promote inflammation, which preferentially uses glycolysis and glutamine catabolism to provide energy (42). Peroxisome proliferator-activated receptor gamma (*PPAR-γ*) expression is significantly reduced in the synovial membranes of RA patients (43). *PPAR-γ* activation inhibits Th17 differentiation by suppressing glutamine catabolism. On one hand, the specific mechanism may involve *PPAR-γ* inhibiting *GLS1* and decreasing 2-hydroxyglutarate (2-HG) levels, thereby regulating lysine demethylase 5 (*KDM5*)-specific trimethylation of Histone H3 at Lysine 4 (H3K4me3) modifications in the promoter and *CNS2* binding regions of the *IL-17* locus. In contrast, *PPAR-γ* inhibits *GLS1* and reduces GSH levels, increases ROS levels, and downregulates retinoic acid-related orphan receptor gamma (*RORγt*) expression (44). In conclusion, *GLS1* may primarily affect FLS, B cell and CD4+ T cell subsets in RA by promoting FLS cell proliferation, inflammatory cell differentiation, and pro-inflammatory cytokine release.

### LIAS

*LIAS* is an iron-sulfur cluster mitochondrial enzyme that replicates the final step of the ab initio pathway that catalyzes lipoic acid biosynthesis, in which lipoic acid is a powerful antioxidant (45). Lipoic acid can be synthesized in the mitochondria by an enzymatic reaction involving octanoic acid. Lipoic acid is essential for mitochondrial α-keto acid dehydrogenase activity and plays an important role in mitochondrial energy metabolism (46). Mitochondria are important organelles in organisms and play several roles, including providing energy to the cell through oxidative phosphorylation and ATP synthesis. When mitochondria produce energy, they store the electrochemical potential energy in the inner mitochondrial membrane. On both sides of the inner membrane, an asymmetric distribution of protons and other ion concentrations results in the mitochondrial membrane potential. Glycolysis oxidizes pyruvate and combines it with coenzyme A, a reaction coupled with the reduction of NAD^+^, to produce CO_2_ and acetyl coenzyme A. Acetyl coenzyme A can enter the tricarboxylic acid cycle, which produces ATP (or GTP), more CO_2_, FADH_2_, and NADH (47). NADH is then involved in the electron transport chain and oxidative phosphorylation. Oxidative stress is an important factor in mitochondrial dysfunction that leads to RA injury and RA-related atherosclerosis (48). *LIAS* is primarily associated with oxidative stress, inflammation, and RA. Significantly lower LIAS expression in mice after LPS induction is accompanied by enhanced inflammatory response and tissue damage (49). LIAS overexpression in experimental atherosclerotic mice significantly increases the number of Tregs and reduces T-cell infiltration (50). Similarly, reduced liver* LIAS* in mice with hepatic fibrosis is accompanied by mitochondrial dysfunction and morphological abnormalities, including mitochondrial edema, reduced density or vacuolization of mitochondrial cristae and matrix, reduced activity of mitochondrial complexes I, II, IV, and V, increased mitochondrial fission activity, and reduced mitochondrial fusion activity (51). Overexpression of *LIAS* reduced hepatic oxidative stress in non-alcoholic fatty liver disease in mice and protected mitochondrial function by upregulating the nuclear factor erythroid 2–related factor 2 to reduce ROS production (52), attenuated the chronic inflammatory response, inhibited NF-kB activity in lung fibrosis in mice (53), significantly increased Treg cell numbers, and reduced T cell infiltration (50). Mutations in *LIAS* stabilize *HIF-1a* in its non-hydroxylated form and promote *HIF-1* activation by inhibiting the activity of prolyl hydroxylases (PHDs), which potentially leads to enhanced glycolytic effects in cells (54). Therefore, *LIAS* and *HIF-1* may be involved in RA progression.

### DLAT

E4 transcription factor 1 (*E4F1*) is a crucial gene involved in controlling mitochondrial function and cell cycle checkpoints that can interact with RA *via P53* (55, 56). *E4F1* regulates *DLAT*. These two factors may synergistically regulate the pathogenesis of RA (57). Mitochondrial PDHC is primarily involved in pyruvate oxidation and the TCA cycle, and provides energy to the body (57). Sirtuin 4 (*SIRT4*) has enzymatic hydrolytic activity and it was significantly downregulated and markedly correlated positively with anti-cyclic citrullinated peptide (anti-CCP) antibody, ESR, and C-reactive protein (CRP) levels in patients with RA (58, 59). *SIRT4* can hydrolyze the lipoamide cofactors of *DLAT*, thereby inhibiting PDH activity (59). In addition, component 1 Q subcomponent-binding protein (C1QBP) in the mitochondria is associated with histological inflammation scores in RA. It can regulate mitochondrial metabolism by affecting PDGH activity through binding to *DLAT* (60, 61). Therefore, *DLAT* may influence the development of RA primarily by affecting pyruvate oxidation in PDHC, the TCA cycle, and mitochondrial function.

### FDX1

*FDX1* is a member of the ferredoxin family, which comprises iron-sulfur (Fe/S) proteins (62). The transcription factors *c-Jun* and *SF1* can synergistically promote the transcription and expression of *FDX1* (63). *FDX1* influences immune cells (dendritic cells, monocytes, macrophages, and iTreg cells) (64). Monocytes in RA prefer to use fatty acid oxidation to provide energy and drive receptor activator of nuclear factor kappa-B ligand (RANKL)-induced osteoclast survival and the associated bone destruction (65).FDX1 was found to significantly promote ATP production in these cells. *FDX1* knockdown significantly promotes production of fructose 6-phosphate, thus affecting downstream glycolysis, and decreases the levels of many long-chain fatty acids, indicating that it promotes fatty acid oxidation (64).

Abnormalities in and regulation of steroid production play an important role in RA. For example, there are multiple abnormal steroid-related metabolites in patients with RA (66). Increased pro-inflammatory factors in RA may be associated with the reduced renal clearance of steroids (67). *FDX1* may be involved in RA development, by potentially influencing this process. Ferredoxin reductase transfers electrons from nicotinamide adenine dinucleotide phosphate (NADPH) to *FDX1*, reducing members of the mitochondrial cytochrome P450 protein family such as cytochrome P450 11A1 (*CYP11A1*) and *CYP11B* (62). *CYP11A1* catalyzes the conversion of cholesterol to pregnenolone *via* side-chain cleavage in the mitochondria, which is the rate-limiting step in adrenal steroid biosynthesis (63). *CYP11B* promotes the conversion of cortisol to corticosterone, or aldosterone (62). *CYP11A1* also converts vitamin D3 to the non-calcemic analog 20S-hydroxyvitamin, which significantly reduces the release of pro-inflammatory T cell subsets and pro-inflammatory cytokines, increases the proportion of Treg cells, and improves symptoms in a mouse model of arthritis (68).

### MTF1

*MTF1* is a classical metal-binding transcription factor closely associated with copper homeostasis in eukaryotic organisms (69). Copper loading induces transcriptional activation of metallothionein (*MT*) through *MTF1* and metal responsive element (MRE)-dependent pathways and promotes the nuclear expression of *MTF1*, which promotes metallothionein expression (70). When copper is depleted, *MTF1* also binds to the MRE of *CTR1B* to promote its transcription and expression of *CTR1B*, facilitating the introduction of copper to maintain copper homeostasis (71). In addition to MTF1 to maintain copper homeostasis, mammalian cells express a variety of copper transporter proteins or enzymes, such as copper transporter 1 (CTR1), cytochrome c-oxidase 1 (Cox1), Cox2, Cox11, Cox17, synthesis of cytochrome c oxidase 1 (Sco1), Sco2, superoxide dismutase 1 (SOD1), antioxidant-1 (Atox1), ATPase copper transporting alpha (ATP7A), ATPase copper transporting beta (ATP7B), extracellular superoxide dismutase (ecSOD, SOD3), and lysyl oxidase (LOX). Copper homeostasis can be divided into several stages. Firstly, CTR1 uptake of copper, where the copper is transported *via* protein interactions to three different sites for further processing. For example, ligand-bound copper ions and copper transport proteins, such as Cox1, Cox2, Cox11, and Cox17, are subsequently transported to Sco1 and Sco2 in mitochondria (72) whereas in the cytoplasmic lysates and mitochondrial gap copper is transported to SOD1 (72). Copper is transported *via* ATP7A or ATP7B to the secreted enzymes EcSOD, SOD3, and LOX (72). Other copper transporter proteins and their specific roles have been clearly described, and here we focus only on cuproptosis-related genes (72).

MTF-1 can directly or indirectly regulate a variety of cellular functions, and is mainly associated with hypoxic conditions in patients with RA. The RA risk SNP (*rs28411362*) forms a 3D contact with the *MTF1* promoter during inflammatory factor-stimulated chromatin remodeling of RA FLS, whose binding motif stimulates FLS recruitment, and *MTF1* inhibition significantly suppresses FLS cytokine and chemokine production and improves the mouse arthritis model (73). Under hypoxic conditions, MTF-1 expression promotes the transcriptional activation of phosphatidylinositol glycan anchor biosynthesis class F(*PIGF*) to promote angiogenesis and enhance endothelial growth and permeability *via* the vascular endothelial growth factor (*VEGF)* (74). MTF1 also promotes the activity of hypoxia-inducible factor-1 (*HIF-1*) (75). *HIF-1a* is a major regulator of cells under hypoxic conditions and is highly expressed in the RA synovium (10, 76, 77). *HIF-1a* can also induce MMP-3 production to promote bone destruction (10). *HIF-1a* promotes pro-inflammatory T cell arrest in joints and Th17 differentiation through transcriptional activation of *RORγT* and tertiary complex formation with RORγt and p300 recruitment to the *IL-17* promoter. *HIF-1* inhibits Treg development by targeting forkhead box P3 (*FOXP3*) for proteasomal degradation (77). *HIF-1a* promotes the conversion of pyruvate to lactate by increasing *LDHA* activity. High concentrations of lactate promote cell proliferation of FLS (10, 76, 77). Furthermore, in addition to its effects on RA FLS, high lactate concentrations can promote pro-inflammatory T-cell arrest in the joints. It is worth noting that *MTF1* responds to copper stimulation through different binding genes (78), and phosphorylation of *MTF1* is essential for the functional activation of MTF (79). For example, *MTF1* promotes *ATP7B* expression by binding to the MRE in the promoter region of *ATP7B* to promote Wilson’s disease caused by copper overload (80). Phosphorylation of the kinase *LATS* of the Hippo pathway and inhibition of *MTF1* protects cells from heavy metal-induced cytotoxicity (81). Thus, *MTF1* primarily responds to excess copper levels in RA, and the hypoxic environment affects multiple pathological aspects of RA.

### CDKN2A

The fraction of cells expressing p16 (*CDKN2A*) is a typical marker of cellular senescence (82). Cellular senescence has been associated with RA in various cell types. For example, senescent T cells are highly inflammatory, secrete cytotoxic mediators, and express natural killer receptors (NKR), bypassing their antigenic specificity (83, 84). Histone deacetylase1 (HDAC1) is overexpressed in RA FLS and promotes cell proliferation in FLS (85). The deacetylase (HDA) inhibitor FK228 inhibits joint swelling, synovial inflammation, and bone destruction in mice with experimentally induced arthritis. It also inhibits the proliferation of RA FLS *in vitro* by a mechanism that involves FK228, thereby inducing high histone acetylation and DKN2A expression in synovial cells, upregulating p21, and decreasing the release of TNF and IL-1β (86). However, it is noteworthy that the senescent phenotype of RA FLS highly expresses CDKN2A and releases more pro-inflammatory mediators in response to TNF or oxidative stress stimuli to promote inflammation (87). The histone methyltransferase EZH2 is strongly induced in chronic inflammation of RA FLS, which may suppress CDKN2A expression and thus contribute to the abnormal response to FLS (88). In addition to its potential effects on RA FLS, CDKN2A may affect RA by influencing the function of macrophages, T cells, and leukocytes. Oxidized low-density lipoprotein (*ox-LDL*) activates multiple immune cells in RA to promote the secretion of pro-inflammatory mediators and assemble Abs to promote the production of immune complexes to mediate RA pathological progression (89). *Ox-LDL* promotes the secretion of *TNF-α* and *IL-1β* by macrophages and functions *via* the *MEG3/miR-204/CDKN2A* axis (90). CDKN2A expression in macrophages inhibits LPS-induced IL-6 production by a specific mechanism involving CDKN2A, promoting ubiquitin-dependent degradation of IRAK1 and impairing the activation of AP-1 (91). Reduced expression of *CDKN2A* in leukocytes appears to be associated with increased CD14++CD16++ monocyte subsets, increased immune complex responses, and overproduction of pro-inflammatory factors in RA (2, 92). EZH2 is also thought to be essential for B and T cell development, and IL-17 in RA patients with RA synovial fluid may inhibit EZH2 expression downregulation in CD4+ T cells and suppress Treg differentiation (93). EZH2 also suppresses CDKN2A expression in naive CD8+ T cells by reducing H3K27me3 levels at two loci (50) and by controlling B-cell maturation (94). Therefore, EZH2 may work in combination with CDKN2A to regulate abnormal T and B cell responses in RA.

### LIPT1

*LIPT1* primarily encodes LIPT1, which catalyzes the transfer of lipoic acid from the H protein of the glycine cleavage system to the E2 subunit of 2-ketoacid dehydrogenase, an essential step in lipid acylation (95, 96). LIPT1 is primarily responsible for regulating glutamine metabolism to support mitochondrial respiration, the TGA cycle, and fatty acid production (95). Mutations in *LIPT1* impair mitochondrial proteolipid acylation and TGA cycling, and promote the accumulation of lactate and pyruvate (95). Among them, lactate and pyruvate can stimulate synovial cell proliferation, angiogenesis, and vascular opacification in patients with RA (97). Little research has been conducted on *LIPT1* in diseases, and further studies are still needed.

## Conclusion

**Figure 1:**
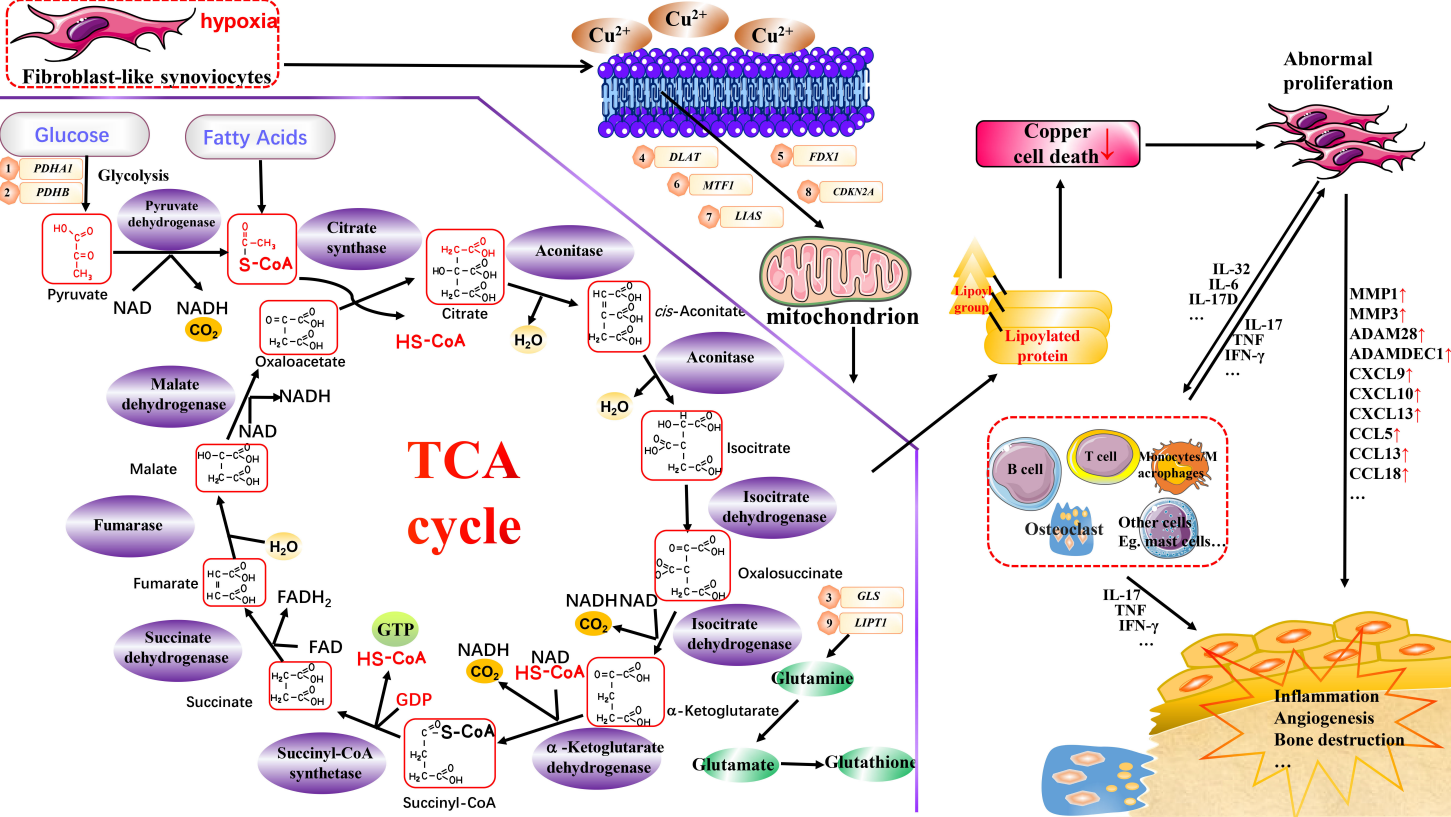
Potential association of cuproptosis and cuproptosis–related genes with RA. Copper can induce cuproptosis by binding to lipid-acylated TCA cycle components, promoting lipid-acylated protein aggregation, and inducing protein stress. This may affect various cells in RA, such as FLS and monocytes/macrophages, thereby aiding in inflammation, angiogenesis, and the bone destruction processes. Vital regulatory genes for cuproptosis are potentially linked to RA through distinct biological functions. However, the specific mechanisms require further investigation.

A specific concentration of copper in an organism contributes to organismal homeostasis. However, the imbalance in copper homeostasis may affect the organism by triggering cuproptosis, leading to disease development. Cuproptosis is considered a potential therapeutic option for oncological diseases, and its possible association with RA is multifaceted (**Figure 1**). First, cuproptosis in multiple immune cells may be suppressed, and this suppression contributes to their over-proliferation in RA. Secondly, several essential regulatory genes of cuproptosis have been identified to be associated with multiple RA processes, such as aberrant FLS proliferation and inflammatory processes in various immune cells. *PDHA1* regulates glycolysis and inflammation; miRNAs primarily regulate *PDHB*, *GLS1*, and *LIPT1* regulate glutamine metabolism; *DLAT* regulates mitochondrial function and the TCA cycle metabolism; and *FDX1* regulates fatty acid oxidation and steroidogenesis; *MTF1* and *LIAS* regulate copper homeostasis; and *HIF-1* and *CDKN2A* regulate cellular senescence. Finally, it is worth noting that cuproptosis is a newly characterized form of cell death, and its specific mechanisms and effects on disease are not as well studied as other forms of cell death, such as apoptosis and ferroptosis. Well-designed preclinical experiments and clinical trials are still required for in-depth studies of cuproptosis and its associated genes in the context of RA, which still present a significant challenge. However, it is undeniably a research direction with great potential.

## Author contributions

JZ is responsible for the collection, collation, and writing of the original manuscript. SG, SS, and DH are responsible for the concept development, revision, and manuscript review. All authors reviewed and accepted the final version.

## Funding

This work was funded by the National Natural Science Funds of China (82074234 and 82071756), National Key Research and Development Project (2018YFC1705200 and 2018YFC1705203), Shanghai Chinese Medicine Development Office, National Administration of Traditional Chinese Medicine, Regional Chinese Medicine (Specialist) Diagnosis and Treatment Center Construction Project-Rheumatology, State Administration of Traditional Chinese Medicine, National TCM Evidence-Based Medicine Research and Construction Project, Basic TCM Evidence-Based Capacity Development Program, Shanghai Municipal Health Commission, and East China Region-based Chinese and Western Medicine Joint Disease Specialist Alliance.

## Conflict of interest

The authors declare that the research was conducted in the absence of any commercial or financial relationships that could be construed as a potential conflict of interest.

## Publisher’s note

All claims expressed in this article are solely those of the authors and do not necessarily represent those of their affiliated organizations, or those of the publisher, the editors and the reviewers. Any product that may be evaluated in this article, or claim that may be made by its manufacturer, is not guaranteed or endorsed by the publisher.

## Abbreviations

RA, rheumatoid arthritis; TCA cycle, tricarboxylic acid cycle; NSAIDs, non-steroidal anti-inflammatory drugs; FLS, fibroblast-like synoviocytes; FDX1, ferredoxin 1; LIAS, lipoic acid synthetase; LIPT1, lipoyltransferase 1; DLD, dihydrolipoamide dehydrogenase; DLAT, drolipoamide S-acetyltransferase; PDHA1, pyruvate dehydrogenase E1 subunit alpha 1; PDHB, pyruvate dehydrogenase E1 subunit beta; MTF1, metal-regulatory transcription factor-1; GLS, glutaminase; CDKN2A, cyclin-dependent kinase inhibitor 2A; MMPs, matrix metallopeptidases; IL, interleukin; CCL20, chemokine (C-C motif) ligand 20; FoxO3, forkhead box O-3; PDHC, the pyruvate dehydrogenase (PDH) complex; ATP, adenosine triphosphate; RUNX2, RUNX family transcription factor 2; PKB, phosphorylated protein kinase B; HK2, hexokinase 2; PDHK1, PDH kinases 1; SIRT6, sirtuin 6; PI3K, phosphatidylinositol 3−kinase; AKT, protein kinase B; ERK, the extracellular signal-regulated kinase; NF-κB, nuclear factor-κB; TNFα, tumor necrosis factor α; NLRP3, the nucleotide-binding oligomerization domain (NOD)-like receptor pyrin domain containing 3; MEG3, maternally expressed gene 3; miR, miRNA; GRP78, glucose-regulated protein 78; ATF6, activating transcription factor 6; CHOP, C/EBP homologous protein; NEK10, NIMA-related kinase 10; IFN-γ, interferon gamma; CCR6, chemokine (C-C motif) receptor 6; CXCR3, C-X-C chemokine receptor 3; PPAR-γ, peroxisome proliferator- activated receptor gamma; 2-HG, 2-hydroxyglutarate; KDM5, lysine demethylase 5; H3K4me3, trimethylation of Histone H3 at Lysine 4; ROS, reactive oxygen species; RORγt, retinoic acid-related orphan receptor gamma t; E4F1, E4 transcription factor 1; SIRT4, sirtuin 4; anti-CCP, the antibodies cyclic citrullinated peptides; ESR, erythrocyte sedimentation rate; CRP, C-reactive protein; C1QBP, component 1, q subcomponent-binding protein; Fe/S, iron-sulfur; RANKL, receptor activator of nuclear factor kappa-B ligand; NADPH, nicotinamide adenine dinucleotide phosphate: CYP11A1, cytochrome P450 11A1; MRE, metal responsive element; PIGF, phosphatidylinositol glycan anchor biosynthesis class F; HIF-1, hypoxia-inducible factor-1; FOXP3, forkhead box P3; PHDs, prolyl hydroxylases; ox-LDL, oxidized low-density lipoprotein; NKR, natural killer receptors.

## Glossary

**Table:**

RA	rheumatoid arthritis
TCA cycle	tricarboxylic acid cycle
NSAIDs	non-steroidal anti-inflammatory drugs
FLS	fibroblast-like synoviocytes
FDX1	ferredoxin 1
LIAS	lipoic acid synthetase
LIPT1	lipoyltransferase 1
DLD	dihydrolipoamide dehydrogenase
DLAT	drolipoamide S-acetyltransferase
PDHA1	pyruvate dehydrogenase E1 subunit alpha 1
PDHB	pyruvate dehydrogenase E1 subunit beta
MTF1	metal-regulatory transcription factor-1
GLS	glutaminase
CDKN2A	cyclin-dependent kinase inhibitor 2A
MMPs	matrix metallopeptidases
IL	interleukin
CCL20	chemokine (C-C motif) ligand 20
FoxO3	forkhead box O-3
PDHC	the pyruvate dehydrogenase (PDH) complex
ATP	adenosine triphosphate
RUNX2	RUNX family transcription factor 2
PKB	phosphorylated protein kinase B
HK2	hexokinase 2
PDHK1	PDH kinases 1
SIRT6	sirtuin 6
PI3K	phosphatidylinositol 3−kinase
AKT	protein kinase B
ERK	the extracellular signal-regulated kinase
NF-κ;B	nuclear factor-κ;B
TNFα	tumor necrosis factor α
NLRP3	the nucleotide-binding oligomerization domain (NOD)-like receptor pyrin domain containing 3
MEG3	maternally expressed gene 3
miR	miRNA
GRP78	glucose-regulated protein 78
ATF6	activating transcription factor 6
CHOP	C/EBP homologous protein
NEK10	NIMA-related kinase 10
IFN-γ	interferon γ
CCR6	chemokine (C-C motif) receptor 6
CXCR3	C-X-C chemokine receptor 3
PPAR-γ	peroxisome proliferator- activated receptor γ
2-HG	2-hydroxyglutarate
KDM5	lysine demethylase 5
H3K4me3	trimethylation of Histone H3 at Lysine 4
ROS	reactive oxygen species
RORγt	retinoic acid-related orphan receptor γt
E4F1	E4 transcription factor 1
SIRT4	sirtuin 4
anti-CCP	the antibodies cyclic citrullinated peptides
ESR	erythrocyte sedimentation rate
CRP	C-reactive protein
C1QBP	component 1 q subcomponent-binding protein
Fe/S	iron-sulfur
RANKL	receptor activator of nuclear factor κ-B ligand
NADPH	nicotinamide adenine dinucleotide phosphate
CYP11A1	cytochrome P450 11A1
MRE	metal responsive element
PIGF	phosphatidylinositol glycan anchor biosynthesis class F
HIF-1	hypoxia-inducible factor-1
FOXP3	forkhead box P3
PHDs	prolyl hydroxylases
ox-LDL	oxidized low-density lipoprotein
NKR	natural killer receptors
